# Towards enabling a cardiovascular digital twin for human systemic circulation using inverse analysis

**DOI:** 10.1007/s10237-020-01393-6

**Published:** 2020-10-16

**Authors:** Neeraj Kavan Chakshu, Igor Sazonov, Perumal Nithiarasu

**Affiliations:** Biomedical Engineering Group, Zienkiewicz Centre for Computational Engineering, College of Engineering, Swansea University, Swansea, SA2 8PP UK

**Keywords:** Inverse analysis, Deep learning, Digital twin technology, Systemic circulation, Blood flow, Aneurysm detection

## Abstract

An exponential rise in patient data provides an excellent opportunity to improve the existing health care infrastructure. In the present work, a method to enable cardiovascular digital twin is proposed using inverse analysis. Conventionally, accurate analytical solutions for inverse analysis in linear problems have been proposed and used. However, these methods fail or are not efficient for nonlinear systems, such as blood flow in the cardiovascular system (systemic circulation) that involves high degree of nonlinearity. To address this, a methodology for inverse analysis using recurrent neural network for the cardiovascular system is proposed in this work, using a virtual patient database. Blood pressure waveforms in various vessels of the body are inversely calculated with the help of long short-term memory (LSTM) cells by inputting pressure waveforms from three non-invasively accessible blood vessels (carotid, femoral and brachial arteries). The inverse analysis system built this way is applied to the detection of abdominal aortic aneurysm (AAA) and its severity using neural networks.

## Introduction

Data-driven analysis and monitoring are key drivers for the evolution of state-of-the-art health care. With increase in patients requiring medical care (Baker 2020), data generation and accumulation is also increasing at an alarming rate (Dinov 2016). This increase has put additional stress on an already challenging medical cyber-infrastructure. In addition to data accumulation, increase in number of patients is also causing unprecedented delays in critical care, as a result of rise in waiting lists. It is also important to note that late diagnosis is a significant reason for medical complications. Since a significant number of diagnosing and monitoring tools employed in critical care are invasive in nature, time-consuming, expensive and labour-intensive, rise in these delays has become inevitable. This has given rise to a need for alternative but robust diagnosis and monitoring tools that can non-invasively detect medical conditions or diseases and help to monitor them with minimal amount of data. A potential key towards development of such tools is artificial intelligence, owing to its capacity to perform complex analyses.

**Fig. 1:**
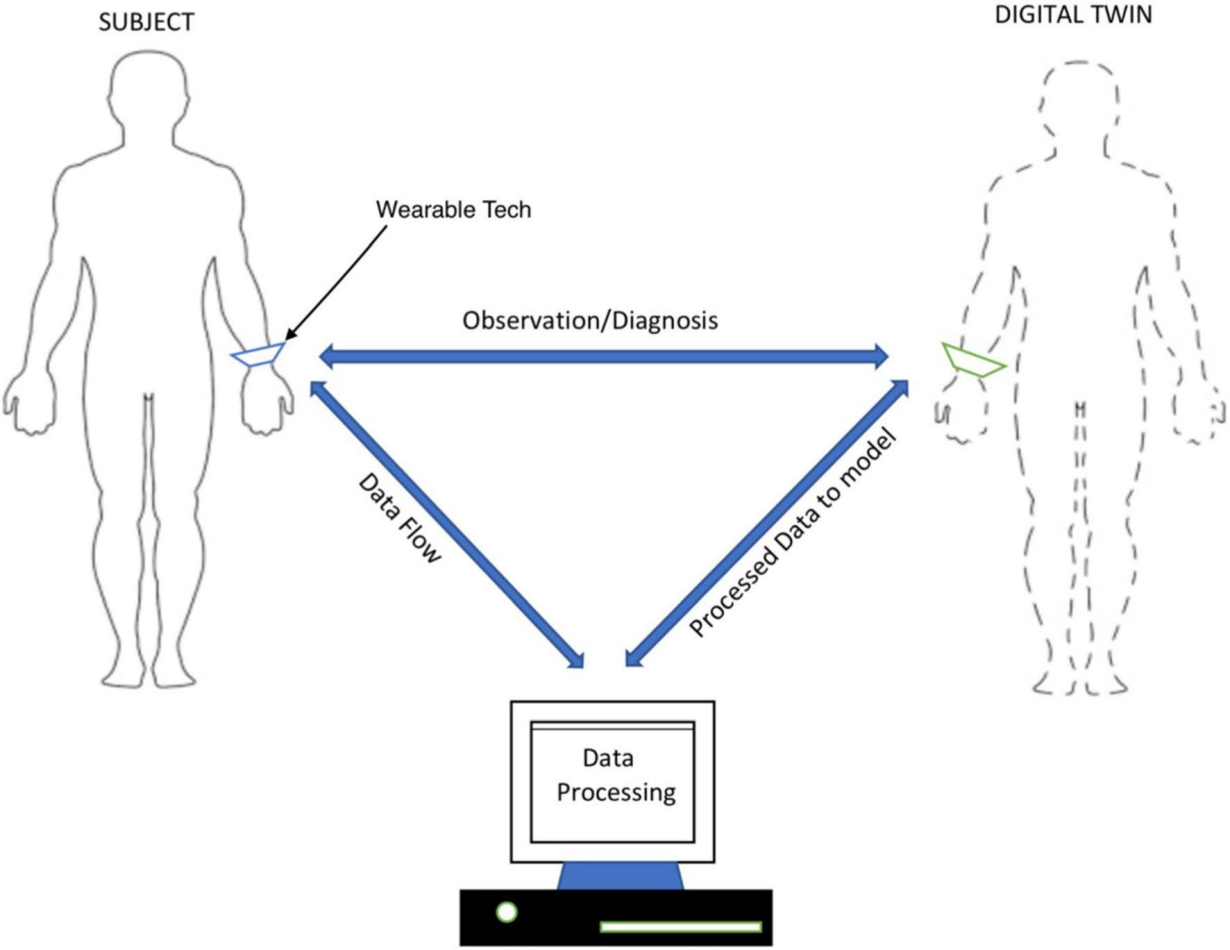
A schematic representation of envisaged digital twin

With recent advancements in artificial intelligence, especially in the area of deep learning, medical applications are being extensively developed with the help of neural networks and other deep learning algorithms. Many of these applications have shown promising results (Bali et al. 2019) and are indicating a future of better and faster tools that will not only detect and monitor medical conditions and diseases but also prevent them before they set in. However, a major issue plaguing this advancement is the treatment of deep learning algorithms as ‘black boxes’. Logical justification of results generated by these algorithms is difficult owing to the complexity of nonlinear transformations involved, leading to growing distrust amongst medical personnel and academicians (Bathaee 2017). Majority of these deep learning algorithms use supervised learning, a method wherein the algorithms are trained using samples obtained from general patient population. This leads to an observation that high chances of misdiagnosis still exist as the analysis is not patient specific. To overcome the above challenges, a digital twin is envisaged as a potential solution as shown in Fig. 1.

A digital twin, virtual representation of an individual, can help perform patient-oriented analysis, using continuous feed of data, thereby increasing the accuracy. This twin can take many forms ranging from investigating only a part of the body to a comprehensive model to study the body in its entirety. The process of making a digital twin can be classified into active, passive and semi-active digital twins. In an active digital twin model, a systemic circulation model is continuously adjusted by continuously monitoring the circulation, at accessible locations and feeding real data into the model as shown in Fig. 1. This type of digital twin has potential applications to diagnosis and monitoring of cardiovascular diseases such as stroke, cardiomyopathy, arrhythmia, aneurysms, stenoses or a combination of these problems occurring at the same time. The passive digital twins are the ones in which we use the data obtained to create an off-line model. This is very common in many of the subject-specific blood flow modelling studies. Some examples of such study include fractional flow reserve (FFR) calculations, understanding rupture potential of aneurysms and stenoses, etc. These passive digital twins can be enhanced to make active or semi-active digital twins by carrying out calculations online while measurements supplied to the underlying model. A semi-active digital twin may have components that have some dynamic nature built in as shown by Chakshu et al. (2019).

Active digital twins work on the principle that the parameters of numerical model being used are updated continuously. This requires constant monitoring of different characteristics a human system for estimating the parameters of the model. In a human systemic circulation, such monitoring will be fast and cost-effective if it can be carried out non-invasively at the peripheral arteries. Since pressure waveforms at the peripheral arteries are easily accessible, determining the waveforms inversely in the remainder of the systemic circulation may provide an easy way of assessing the health of an individual. Thus, inversely estimating the pressure waveforms at various locations of the systemic circulation is key to building an active digital twin. Therefore, an inverse analysis using deep learning is proposed in the present work. Inverse analysis will help in estimating blood pressure waveforms at different locations of the human body by using minimal number of input pressure waveforms. These inversely calculated waveforms can further help in parameter estimations required for updating the systemic circulation model. However, one of the complexities involved in performing inverse calculation or inverse modelling, especially in nonlinear problems, is the potential of obtaining non-unique solutions. However, with the aid of deep learning and appropriate constraints, it is now possible to obtain unique solutions (Tamaddon-Jahromi et al. 2020).

As a demonstration of possible applications of inverse analysis in a cardiovascular system, an example of aortic abdominal aneurysm (AAA) detection and severity classification is also described. An aortic aneurysm is a progressively growing dilation of the aorta with a risk of potentially lethal rupture. Successful treatment of an aneurysm depends on how early it is detected, as the post rupture mortality rate is around 80% (Washington et al. 2016). To detect an AAA in time, a continuous and expensive screening programme is necessary. The existing methods of detection of AAA have several drawbacks. Ultrasound echography is currently considered the most practical and inexpensive modality in AAA screening, but it has limitations due to the fact that the aorta is buried deep in the body. The ultrasound measurement can be obstructed by bowel gas, obesity, calcification and other artefacts (Wilmink et al. 2002). The shape and size of the aneurysm can be determined accurately by 3-D CT or MRI methods but they are substantially more expensive and require injection of a contrast agent. Therefore, they are mainly applied at the latter stages of AAA evolution (Litmanovich et al. 2009; McBride et al. 2015). Besides the aneurysm size, its rupture time depends on mechanical properties of the vessel wall that cannot be determined from the above modalities directly (Vorp 2007). Since the diameter of aneurysm is one of the key factors in assessing the severity of the condition, a neural network model may be used to detect and classify the severity of this problem using computed diameters. Such a classification is one of the several potential applications of inverse analysis, proposed in the present work.

**Fig. 2:**
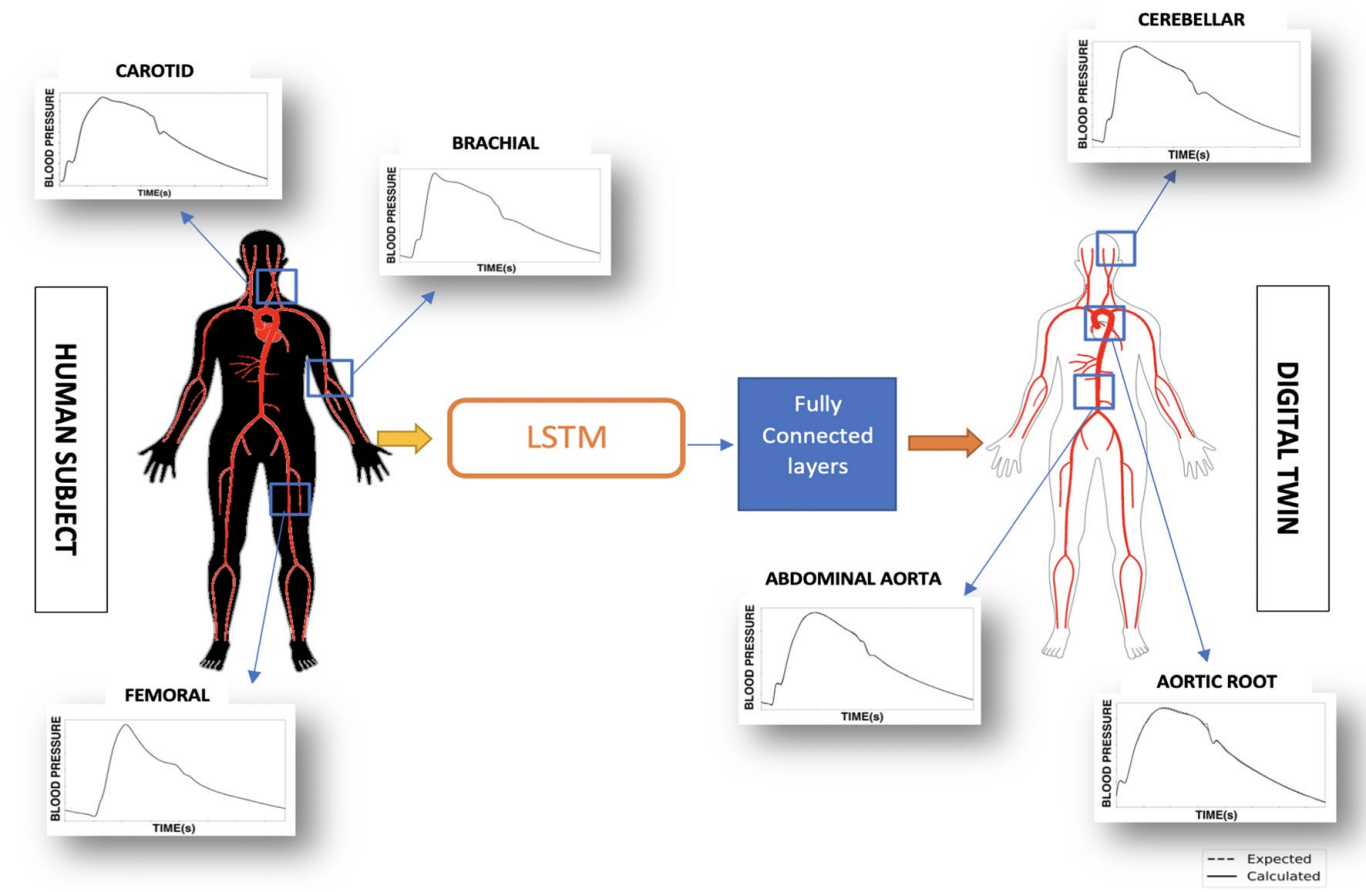
Schematic of inverse analysis to determine waveforms at different locations using three measurements at accessible locations as input

In the present work, three key steps are followed to build and demonstrate the use of deep learning in inverse analysis. Firstly, a database containing computationally generated, realistic blood pressure waveforms is produced using a reduced order model and machine learning. Secondly, a neural network is built and trained to predict unknown blood pressure waveforms using accessible waveforms as input data, as shown in Fig. 2. As seen in the figure, the data inputted at accessible locations (left) is employed to predict pressure waveforms at other locations (right). Finally, an additional neural network is trained to analyse the waveforms predicted by the inverse model to detect abdominal aortic aneurysms (AAA) and their severity.

Briefly, as an essential step to realise the concept of a digital twin, following objectives are proposed. (1) Demonstrate the idea of inverse modelling using the neural networks on human systemic circulations and (2) Propose the idea of using inverse analysis for the detection of an abdominal aortic aneurysm (AAA) and its severity.

## Methodology

In the present work, the primary objective is to develop a system capable of performing inverse analysis on cardiovascular time sequences such as blood pressure waveforms. To demonstrate this, aortic blood pressure waveform, which is a critical parameter to assess the cardiac state of a person, is inversely calculated with the help of deep learning methods. This is carried out using blood pressure waveforms from easily accessible and measurable arteries such as radial, femoral and carotid arteries as inputs.

Supervised learning approach is adopted to perform this deep learning-based inverse analysis. Since the usage of an existing database is a key component to this approach, access to large amounts of data becomes a necessity. However, for biomedical applications, access to large amounts of data means access to thousands of patients and their medical records, which is challenging given the time required and feasibility. To circumvent this problem, in the present work, a method to generate virtual patients, a close approximation of human patients, is proposed. These virtual patients are used to build the database required to train deep learning algorithms. The inverse method demonstrated in the present work may be extended to real patients using wearable technology.

### Virtual patient database

The objective of virtual patient generation for a cardiovascular system is to build a numerical model for blood flow that closely resembles a human patient in terms of blood flow parameters and vascular network. Previous research in virtual patient database generation methods can be found in references (Willemet et al. 2015; Charlton et al. 2019; Huttunen et al. 2019). Two key components are required to build such realistic models, a validated one-dimensional haemodynamic model and realistic arterial networks based on anthropometric and haemodynamic parameters to represent a reliable human cohort.

#### One-dimensional haemodynamic model

The haemodynamic model used in this work, a modified version of model proposed by Mynard and Smolich (2015) considers a vascular network of 123 of the major vessels in the systemic circulation. Only systemic arteries are considered; thus, the systemic veins and pulmonary systems are neglected. The major vessels in the systemic arteries are treated as 1-D vessel segments. A two-chamber 0-D heart model is coupled with inlet of the aorta, while the outlet of peripheral vessels connects to a three-element Windkessel model, which accounts for the micro-circulation. A large number of alternative one-dimensional models can be found in references Mynard and Nithiarasu (2008), Boileau et al. (2015), Carson and Van Loon (2017), Low et al. (2012), Müller and Toro (2013, 2014), Alastruey et al. (2014), Keijsers et al. (2015), Mynard and Smolich (2015), Huang and Müller (2015), Trenhago et al. (2015), Müller et al. (2016), Vassilevski et al. (2020), Bessonov et al. (2016), Simakov and Simakov (2009), Boileau et al. (2017) and Carson et al. (2019b, d).

#### 1-D vascular modelling, heart and connectivity between vessels

Blood flow in the 1-D vessel is governed by the set of nonlinear equations [Eq.  (1)]. A flat velocity profile is assumed for the convective acceleration term, and a profile with a small boundary layer is chosen for the viscous friction term. A viscoelastic constitutive law is chosen for the walls, which consists of a power law model for the elastic term and a Voigt model for the viscous wall term [Eq. (1c)]. The vascular beds in this model are treated using three-element Windkessel models, which are constructed using, (1) lumped compliances on the arterial side, (2) characteristic impedances coupling any number of connecting 1-D arteries to the lumped parameter vascular bed and (3) a constant vascular bed resistance to represent the downstream resistance of the micro-circulation. These vascular bed models are incorporated in all vascular beds except for the liver and myocardium. For a detailed discussion of the vascular bed modelling of liver and myocardium, see Mynard and Smolich (2015).

A 0-D (lumped model) of heart is used in this model. Lagrange multipliers are used to connect 1-D vessels and are used to conserve mass and total pressure at vessel junctions. Conservation of mass and conservation of static pressure are used to connect the 1-D and 0-D models. The system of equations solved using the methodology in Carson and Van Loon (2017) are:

1a $$\begin{aligned} \frac{\partial A}{\partial t} +\frac{\partial Q}{\partial x}&=0 \end{aligned}$$

1b $$\begin{aligned} \frac{\partial Q}{\partial t} +\frac{\partial \left( {Q^2}/{A} \right) }{\partial x}+\frac{A}{\rho } \frac{\partial P}{\partial x}&=-2\pi \gamma \nu \frac{ Q}{A} \end{aligned}$$

1c $$\begin{aligned} P-P_{0}-P_{\mathrm {ext}}&=\frac{2\rho c_{0}^{2}}{b} \left [ \left (\frac{A}{A_0} \right )^{\textstyle \frac{b}{2}}-1 \right ]+\frac{\varGamma }{A_0\sqrt{A}}\frac{\partial A}{\partial t}, \end{aligned}$$

where *Q* is the volumetric flow rate, *P* is the hydrostatic pressure, $P_{\mathrm {ext}}$ is the external pressure, *A* is the lumen cross-sectional area, *t* is the time, *x* is the axial coordinate, $\rho$ is the density of blood, $\nu$ is the kinematic viscosity of the blood, $\gamma$ is the viscosity parameter, $\varGamma$ is the viscoelastic parameter and *c* is the intrinsic wave speed. Subscript 0 represents the stress free condition.

#### Scaling vascular network and cardiac outflow parameters to create realistic human models

Prior to the generation of a synthetic patient database, a personalised model generation process is proposed to realistically and physically represent human systemic circulation. The personalised model of the systemic circulation for a human patient is generated by adapting geometric and haemodynamic parameters using nominal and ordinal inputs, such as gender, age, weight and height. This type of adaptation on the above 1-D model has been validated against in vivo data in previous publications (Carson et al. 2019a, c; Carson and Van Loon 2017).

**Fig. 3:**
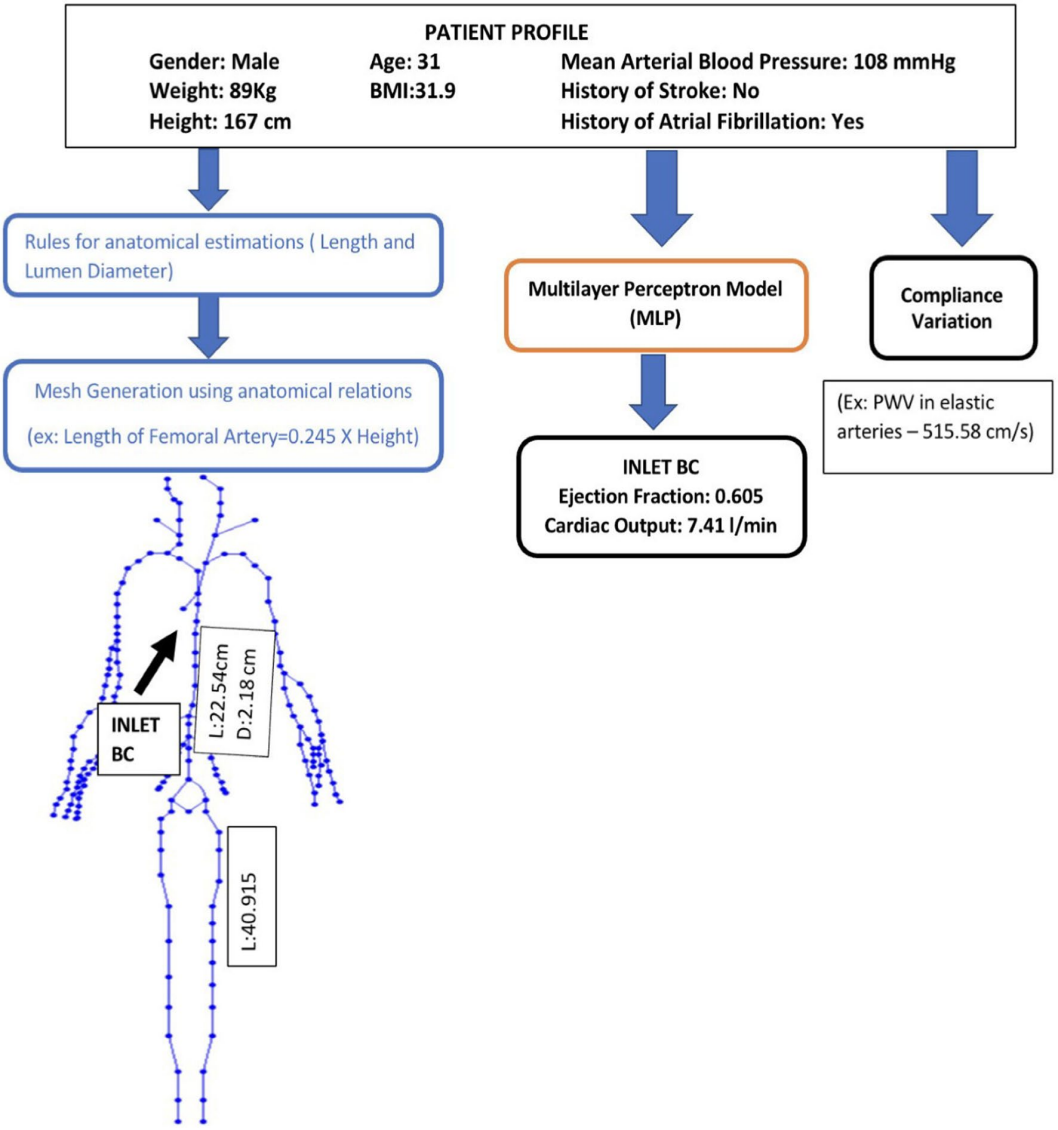
Example of personalised model generation using patient profiles. This includes three parallel workflows: for generation of the geometrical mesh parameters (left), for generation the inlet boundary conditions (centre) and for generation of elastic parameters of the mesh segments

To achieve a realistic representation of arteries in the systemic circulation model, the geometrical parameters of the vascular network used in the above haemodynamic model are scaled using validated empirical relations [such as Eq. (2)], observed or derived by various studies (Passera et al. 2013; Sandgren et al. 1999; Sonesson et al. 1994; Chiaganam et al. 2013) on general population, that are functions of parameters such as weight, height and age (left half of Fig. 3). These relations lead to the geometry and mesh for a patient. Some of the equations used for scaling are:

2a $$\begin{aligned} L_{\mathrm {femoral}}&= 0.245{H} \end{aligned}$$

2b $$\begin{aligned} L_{\mathrm {descending\, aorta}}&=-2.6235+0.1507{H} \end{aligned}$$

2c $$\begin{aligned} D_{\mathrm {abdominal\, aorta}}(\mathrm {begin})&=14.10+0.13{A}\nonumber \\&\quad +(-1.09+0.04{A}){G}+5.8\,\mathrm {BSA}, \end{aligned}$$

where *A* is the age in years, *H* is the height in cm, *G* is the gender (1 for male, 0 for female), $\mathrm {BSA}$ is the body surface area in m$^2$. Parameter ranges of the scaling are described in Sect. 3.1.

**Fig. 4:**
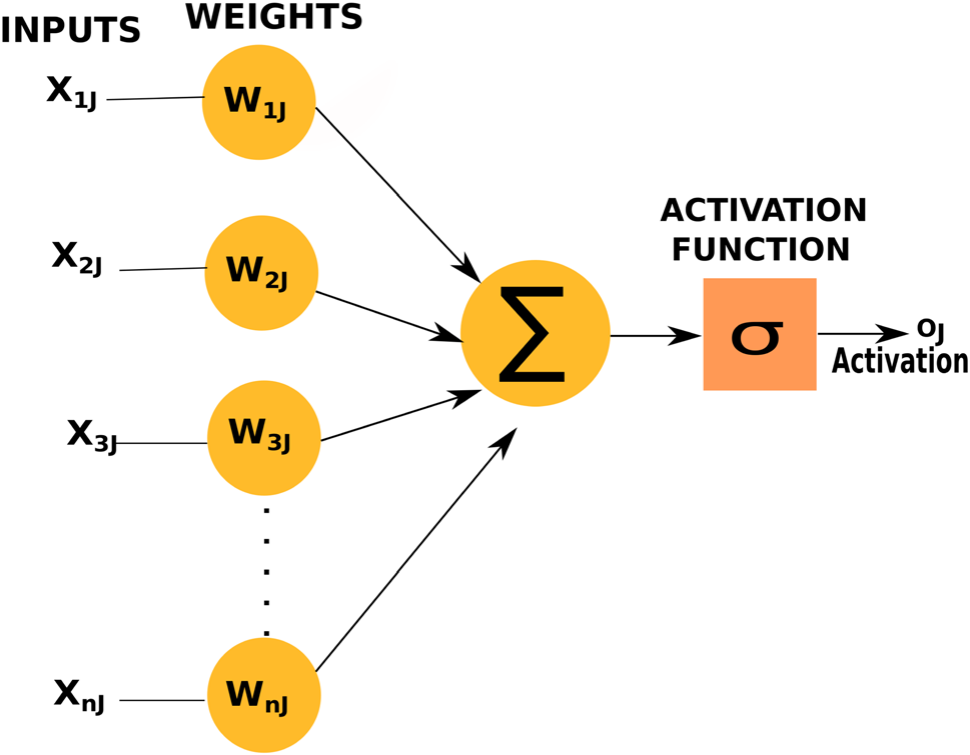
An artificial neuron used in multilayer perceptron model (MLP)

The cardiac outflow parameters such as ejection fraction and cardiac output are used to modify inlet flow boundary conditions at the aortic root. These values are calculated using multilayer perceptron (MLP) models (Haykin 1994), as shown in Fig. 4. Multilayer perceptron model is a type of neural network. Every node of this network represents a connected unit called artificial neuron or perceptron. In Fig. 4, $X_{nj}$ are the discrete input values, $W_{nj}$ are their corresponding weights. The weighted inputs are summed up and the sum is nonlinearly mapped using an activation function $\sigma$ to obtain an output $O_j$. One neural layer confines a certain number of these neurons. A cascaded set-up of several these hidden neural layers, along with an input layer and an output layer, constitutes the neural network.

For calculating inlet flow boundary conditions, the MLP models are trained using a public medical database (Melillo 2015; Goldberger et al. 2000) with inputs such as the mean arterial pressure, pre-existing heart conditions and so on as shown in central workflow in Fig. 3. These MLP models have an architecture of three to four hidden layers, with each layer having the number of neurons varying between 2 and 32, along with an input layer with the number of neurons varying between 4 and 7, and an output layer of one neuron for predicting the value of interest. The hidden layers have ‘TanH’ or hyperbolic tangent as activation function and output layer has either ‘sigmoid’ or ‘ReLU’ (Rectified Linear Unit) depending on the output being predicted. For example, in the case of ejection fraction, since the expected value is between 0 and 1, the sigmoid function is the suitable activation function for the output layer. All models are trained with mean squared error (MSE) as the cost function and ‘Adam’ optimiser (Kingma and Ba 2014).

Haemodynamic parameters of the mesh, such as compliance of the vessels, calculated using pulse wave velocity (PWV), is adjusted for ageing-related changes using equations from literature (McEniery et al. 2005), as shown in the rightmost workflow in Fig. 3.

In order to create a realistic virtual patient database and obtain a closer approximation of actual human patients, the above described personalised model generation is adopted to create patient profiles of the required number of virtual patients, as shown in Fig. 3.

To train machine learning algorithms with better accuracy, virtual patient databases need to be chosen such that it covers a wide range of data, accounting for various combinations of input parameters (age, weight, height, gender, blood pressure and pre-existing heart/medical conditions). The input parameters in personalised model generation, representing anatomical and haemodynamic input parameters of a patient, are referred to as a patient profile in this work (see Fig. 3). Based on required number of virtual patients, a number of such patient profiles are generated by randomising the input parameters, by sampling a uniform distribution of these parameters. Each of these patient profiles is used to generate the required number of virtual patients, each representing a unique cardiovascular system. A sample of one such virtual patient is shown in Fig. 3. Here, the three parallel processes for mesh generation, calculating inlet boundary conditions and haemodynamic parameters, are represented by three workflows. In total, a database of 4137 such healthy virtual patients are generated. More details of generation of the database are presented in Sect. 3.1.

### Application of inverse analysis in abdominal aortic aneurysm (AAA) detection

**Fig. 5:**
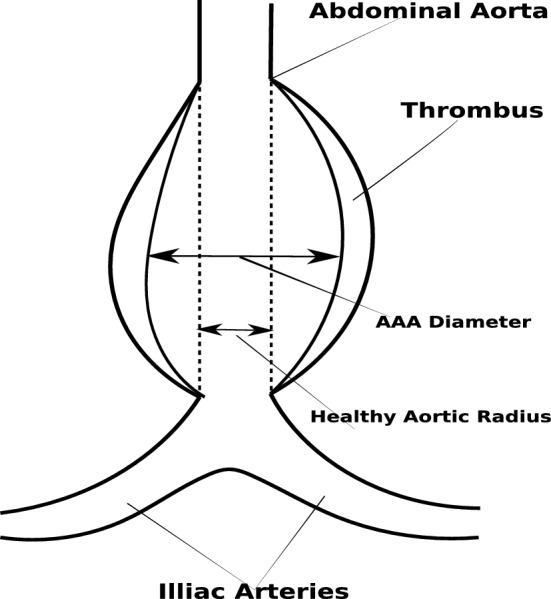
A model of an abdominal aortic aneurysm

Abdominal aortic aneurysm (AAA) mainly occurs above the junction where aorta branches out into iliac arteries (see Fig. 5). Various factors such as ageing and smoking can conduce decrease in the elastin fibres, that can result in a significant increase in vessel wall stiffness in this part of aorta. The enhanced blood pressure, along with the increased vessel stiffness, can cause the expansion of the vessel beyond the elastic deformation, leading to localised increase in diameter of the vessel.

The increase in diameter leads to decrease in the characteristic impedance of arteries, which is an essential parameter for propagation and reflection of pulse waves in the arterial system (Sazonov et al. 2017). The characteristic vessel impedance can be evaluated from the equation,

3 $$\begin{aligned} { Z = \frac{\rho c_0}{A_0} = \frac{4}{\pi } \frac{\sqrt{2\rho \,(\frac{4}{3} Eh)}}{D_0^{5/2}}}, \end{aligned}$$

where *E* is the Young’s modulus, $D_{0}$ is a reference lumen diameter, *h* is the wall thickness. As it is seen from Eq. (3), the characteristic impedance decreases proportionally with $D^{-5/2}$ if the wall stiffness does not change with increase in aneurysm size. Nevertheless, the characteristic impedance can stay constant if the vessel wall stiffness $\frac{4}{3}Eh$ increases dramatically with the aneurysm diameter (of the order of $D^5$).

In vivo measurements of effect of AAA diameter on the wall stiffness appears to show that the wall stiffness can increase with the growth of aneurysm (Sekhri et al. 2004), which is not confirmed by Kolipaka et al. (2016). This makes dependence of $\frac{4}{3}Eh$ on *D* questionable. Some studies (Cebral et al. 2014) indicate that aneurysm ruptures are caused by localised degradation and weakening of the wall. Therefore, at least in a number of critical cases, when the vessel is about to rupture, we could assume that the wall stiffness, and hence the characteristic impedance, at the location of rupture, are almost non-existent. In this case, the aneurysm will lead to a significant wave reflection that can be detected during the waveform analysis.

Virtual patients with AAA are modelled with fusiform aneurysms in the abdominal aorta. The diameter is varied sinusoidally along the length of the aneurysm (Low et al. 2012), with the widest area occurring at mid-length. The aneurysms are classified into small AAA (3–4.4 cm), medium AAA (4.5–5.4 cm) and large AAA (> 5.5 cm) aneurysms. Within each category, diameter for a virtual AAA patient was randomly chosen. However, post database generation, at least one occurrence of all diameters within one decimal place between 3 and 6.9 cm was verified in order to account for every possible diameter. As the wall stiffness is of crucial importance in large aneurysms, it is indirectly modified using pulse wave velocity (PWV). In order to mimic critical cases, where rupture of vessel wall is imminent, in few personalised models the stiffness is made very low by decreasing PWV drastically at randomised locations along the aneurysm. In these locations, local PWV of 5 elements is reduced to values between 14 and 30% of their original PWV. The local stiffness for small and medium AAAs are not modified. This allowed for wide range of possible AAA cases to be simulated.

The virtual AAA patients are added to the database using their generated personalised profile. The range used for the randomised selection of input parameters, required for calculating anatomical and haemodynamic factors, is similar to that of healthy (non-AAA) cases (see Sect. 3.1). Generating all possible combinations of input parameters and AAA sizes is a real challenge. Hence, a randomised selection of their combinations is chosen to generate as many different combinations as possible to include into the database.

In total, a database of 8659 virtual patients is created with 4137 healthy (or non-AAA) cases and 4522 AAA cases.

#### Understanding blood waveforms in AAA

The database generated in Sects. 2.1.3 and 2.2.1 consists of a collection of pressure waveforms, each of which represents a different vascular geometry and aneurysm parameters. In order to give the reader an insight in to the variation of waveforms captured in the database, a detailed analysis of these waveforms is presented in this section. The pulse wave velocity (PWV) in the ascending aorta, an elastic vessel, varies between 5 and 10 m/s, depending on age, and increases towards the peripheral arteries. Analysis of a typical vivo waveform, in the frequency domain, shows that the first 5 harmonic components contain 95% of the pulse energy.

**Fig. 6:**
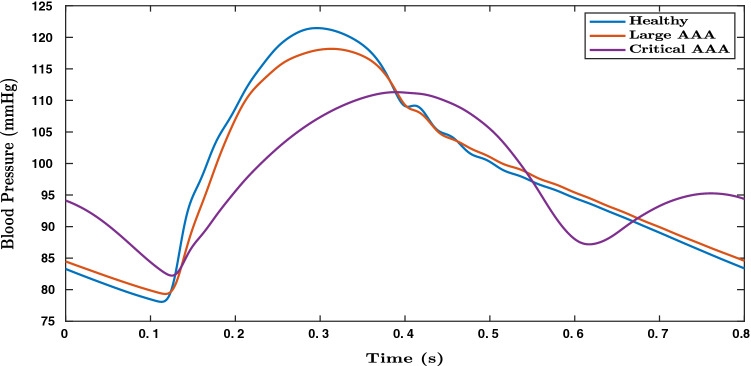
Blood pressure waveforms (simulated) in abdominal aorta computed close to its distal end for healthy, large AAA and critical AAAs (where the vessel is about to rupture) cases

**Fig. 7:**
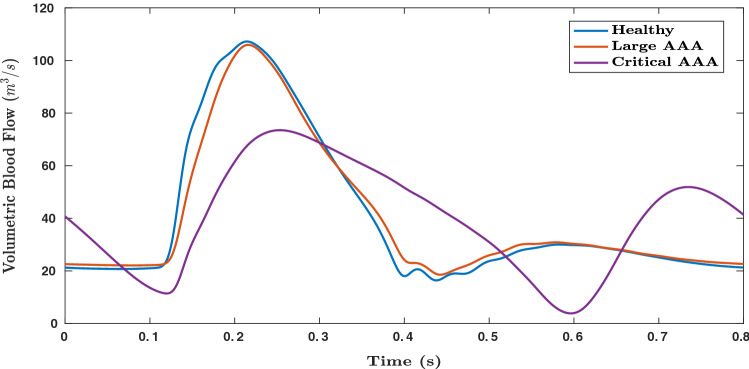
Volumetric blood flow rate (simulated) in in abdominal aorta computed close to its distal end for healthy, large AAA and critical AAA (where the vessel is about to rupture) cases

Figures 6 and 7, respectively, show the variation in pressure and flow rate waveforms for healthy and aneurysmal aortas. Large AAA refers to aneurysms with AAA diameter greater than 5.4 cm and critical AAA refers to large AAA which is about to rupture, with critical points on vessel wall where stiffness is close to zero. In Fig. 7, a small phase shift can be observed in volumetric blood flow rates between healthy, large AAA and critical AAA conditions. These observations make this medical condition a suitable application for deep learning, where parameters can be observed in compounded nonlinear domains. In the present work, only blood pressure waveforms are used for deep learning applications.

### Deep learning for inverse pressure waveform calculation and AAA classification

Deep learning, a type of machine learning, involving multiple layers of nonlinear transformation is the method chosen in this work to perform supervised learning. Major part of deep learning involves usage of artificial neural networks. Basic building blocks of these networks are nodes or connected units called artificial neurons or perceptrons, which incorporate a nonlinear mapping of weighted inputs, as shown in Fig. 4.

**Fig. 8:**
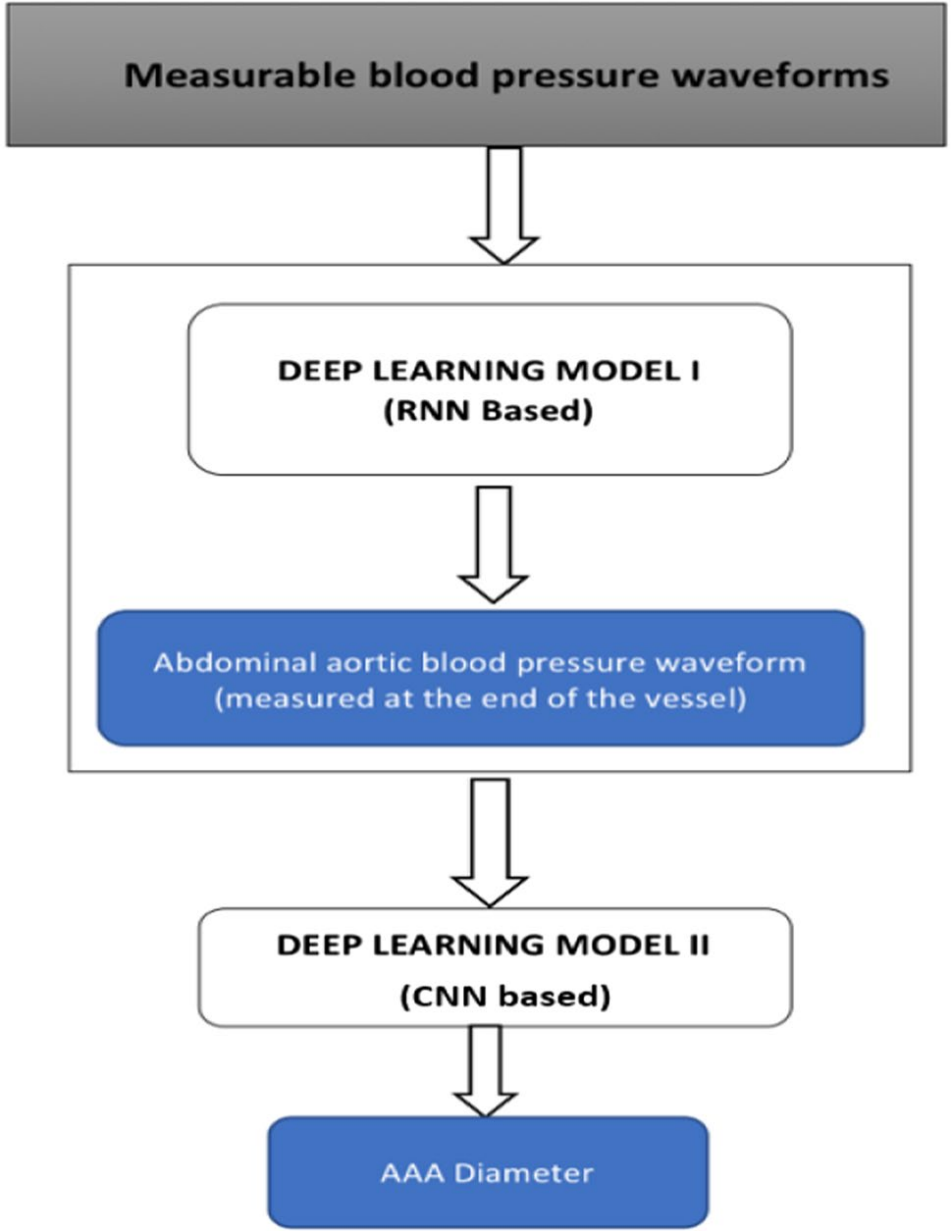
LSTM-based inverse analysis and AAA classification

The patients’ database described in Sect. 2.1 is used to train deep learning algorithms for performing inverse analysis. Upon completion of such an inverse model, deep learning is further used in the development of AAA classification tool to calculate the parameters of interest, which in this example is the diameter, using outputs generated by the inverse model (see Fig. 8). In summary, a deep neural network is first used to determine waveforms at various locations of a blood flow network and then an additional neural network of a different configuration is employed to analyse AAA diameter.

#### Long short-term memory (LSTM)-based neural network for inverse analysis

**Fig. 9:**
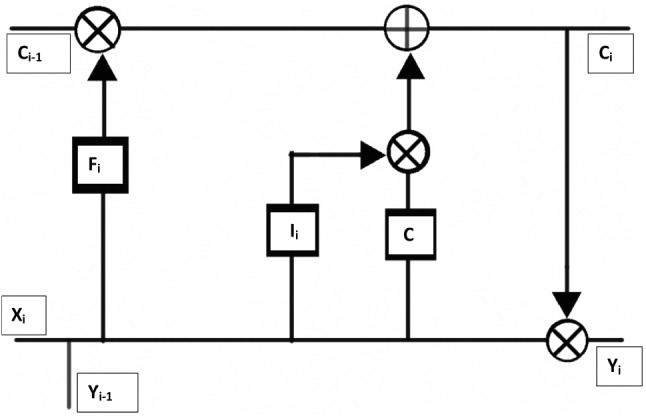
Long short-term memory (LSTM) cell consisting of cell state as well as forget, input and output gates

The primary objective of inverse analysis, shown in Fig. 8, is carried out using a recurrent neural network (RNN) (Rumelhart et al. 1988). RNNs are a form of neural networks designed to handle sequential data, which use a concatenated input consisting of output from the previous step and input from the current time step. Output from the previous time step refers to the prediction made by the RNN cell for input values of the previous time step. Input from the current time step refers to the value present in external input data provided to the RNN cell, at that particular time step. Since the input data of interest is sequential in nature (pressure waveforms from available sites such as carotid, femoral and brachial arteries), an RNN is found to be the most robust and computationally viable option. Amongst the different types of RNNs, the most suitable form is a long short-term memory (LSTM) cell (Hochreiter and Schmidhuber 1997; Gers et al. 1999). Regular RNN cells perform well, especially when the short duration data is processed. However, they fail when this duration increases. This issue is solved in an LSTM cell of the kind shown in Fig. 9 that keeps track of dependencies between elements in the input sequence using a cell state.

A cell state is a parallel flow of data in an LSTM cell. It allows for certain information to be carried in parallel to actual data to track various dependencies between elements. For example, in the context of blood pressure waveforms, prediction of dicrotic notch depends on information from peak blood pressure, predicted several time steps before the notch. Such long term dependencies can be retained in the cell state. In Fig. 9, the cell state ($C_i$) is passed through the upper channel and a concatenated signal, consisting of input from the current step ($X_{i}$) and output from the previous step ($Y_{i-1}$), is passed through the lower channel. Gates $F_{i}$, $I_{i}$, *C*, are used to learn from previous steps and predict the next one. Each of these gates refers to a combination of neural layer connected to the cell state through a logical operator or combination of logical operator and other gates. Based on the logical operation carried at the end of their respective neural layer, gates can be identified for their functions such as to forget, retain, select or modify cell state which in turn affects the output value.

Using blood pressure waveforms measured for one cardiac cycle at three locations, carotids, brachial and femoral arteries, the above neural network model calculates time sequences, which here are the blood pressure waveforms, from various arteries of the body, some of which are located upstream. It is observed that only one or two input blood pressure waveforms, with different combinations between carotid, femoral and brachial arteries, failed to predict with the same level of accuracy as that from three inputs. Thus, it can be inferred that a minimum of three input blood pressure waveforms and their time sequences are required to achieve acceptable levels of accuracy. The neural network architecture used is detailed in Table 1. The model is compiled with mean squared error (MSE) as the cost function and trained with ‘Adam’ optimiser, a modified gradient descent method (Kingma and Ba 2014). The data is split into training (80%) and testing data sets (20%). During training, the training data set is further split into training(80%) and validation subsets (20%).

**Table 1:** Architecture of deep learning model I

Type of RNN cell	Long short-term memory
Number of hidden layers	2
Number of cells in hidden layers	32
Number of inputs	3
Number of fully connected layers	1 (output layer)
Cost function	Mean squared error
Optimiser	ADAM
Batch size	300
Number of epochs	1400

#### Convolutional neural network for AAA classification

In AAAs, the blood pressure waveform close to the distal end of abdominal aorta is a superimposed wave that includes reflections and negative pressures. This waveform contains effects of any vessel enlargement. It is important for us to extract these features from the waveform in order to detect and analyse the severity. Traditionally, signal processing techniques such as fast Fourier transforms (Sazonov et al. 2017) and CEPSTRUM analyses are considered as suitable options for such feature extraction. However, with deep learning, these features can be extracted with compounded nonlinear mapping, thereby extracting higher number of features compared to the traditional methods. The most suitable neural network for such application is a convolutional neural network (CNN) (Zubair et al. 2016). CNNs use convolution to extract features from the data. The weights or coefficients in the filters, or convolutional layers, used for these convolutions are trained using a gradient descent-based optimiser to extract the features affecting the parameter of interest, which in this work is the AAA diameter.

**Fig. 10:**
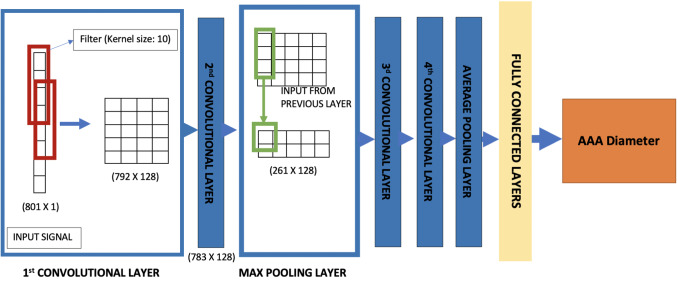
1-D convolutional neural network used to detect severity of AAA

The waveform inversely calculated by the RNN model at a location on the abdominal aorta close to the distal end, about 3 cm downstream, is the input set to a 1-D-CNN (see Fig. 10) classifier which is trained to extract features from the signal and classify the severity of the aneurysm into categories based on the AAA diameter. The categories or classes chosen in this work are healthy (< 3 cm), small AAA (3–4.4 cm), medium AAA (4.5–5.4 cm) and large AAA (> 5.4cm). The architecture of the 1-D CNN model is detailed in Table 2.

The convolutional neural networks employed here utilise convolutional filters in first two layers to capture features from the signal. Convolutional filters use the operation of convolution, multiplication of predetermined weight matrix of a chosen size, to highlight or extract certain aspects in the data provided. Features extracted from them are then compressed using maximum pooling layer, which chooses the maximum amongst extracted features in a given window. To perform with better accuracy, the output from maximum pooling layer is further passed onto another set of convolutional layers before pooling the features using global average (see Fig. 10). The pooled features are then used by fully connected layers, perceptron layers, to estimate the severity of AAA. This network is compiled with ‘categorical cross-entropy’, a combination of softmax function and cross-entropy (Goodfellow et al. 2016), as the cost function. Since classification uses probability distribution, categorical cross-entropy uses softmax function, i.e.

4 $$\begin{aligned} f(s)_{i}=\frac{\hbox{e}^{s_{i}}}{\varSigma ^{C}_{j=1}\hbox{e}^{s_{j}}}, i= 1,\ldots , C \end{aligned}$$

to calculate a probability distribution. Here, *s* are the output scores, a value in the range of 0 to 1, calculated by the neural network for each of the *C* number of categories. Equation 4 is used to calculate loss as

5 $$\begin{aligned} {\text{CE}}=-\varSigma ^{C}_{i}t_{i}\log (f(s)_{i}) \end{aligned}$$

Here, CE is the cross-entropy with $t_{i}$ being the target or expected probabilities and $f(s)_{i}$ is the calculated probabilities. In training, the data is split into training (80%) and testing data sets (20%). Stratified *K*-fold cross-validation method is used to train and evaluate the model. In *K*-fold cross-validation method, the data is split into *K* subsets, and the model is iteratively trained by holding out one of the subsets as the validation set. Each subset is marked as the validation set only once during the iterations. Here, the number of folds, *K*, is 10. This approach is adopted in order to mitigate the problem of overfitting, as it allows for tuning of hyperparameters within the training data set by iteratively holding out one of the ten folds as validation set. The only difference between *K*-fold cross-validation and stratified *K*-fold cross-validation is that the subsets are chosen such that there is equal representation of data from each of the categories in a multi-class classification.

**Table 2:** Architecture of deep learning model II

Type of neural network	Convolutional neural network (CNN)
Number of hidden layers	8
Number of filters	128 (1st and 2nd convolutional layers)
	256 (3d and 4th convolutional layers)
Kernel sizes (convolutional layers)	10 (all layers)
Number of pooling layers	2 (1 max pooling and 1 average pooling)
Number of fully connected layers	3
Number of cells in fully connected layers	16, 8, 4
Number of input sequences	1

## Results and discussion

In this section, results observed for inverse analysis using LSTM-based neural network (RNN) and the example of application of inverse analysis to detection of aortic abdominal aneurysm (using CNN) are presented and discussed in some detail. A brief analysis of the above generated virtual database is also presented to give the readers an overview of the baseline characteristics amongst the virtual patients.

### Analysis of the virtual database

The virtual database generated consisted of a total 8516 patients, with 4137 healthy cases and 4392 AAA cases.

**Table 3:** Baseline characteristics of virtual patients (without AAA) used in the virtual database

	Average [95% CI] [$n=4137$] ($\hbox {Females}=1734$)	Minimum value	Maximum value
Age (years)	61.39 [60.87–61.90]	30.00	89.00
Weight (kg)	96.11 [95.62–96.59]	40.00	120.00
Height (cm)	174.94 [174.47–175.41]	150.00	200.00
Ejection fraction (%)	61.82 [61.74–61.91]	57.00	66.99
Pulse pressure (mmHg)	53.46 [53.09–53.83]	28.08	87.81
Cardiac output (L/min)	5.72 [5.70–5.75]	4.50	7.00
Pulse wave velocity (m/s)	9.20 [9.16–9.23]	6.80	11.96

Table 3 shows the average values as well as minimum and maximum values seen in virtual database for the healthy patients. The pulse wave velocity (PWV) recorded in this table refers to heart-femoral pulse wave velocity. The average value of PWV and different parameters in the 1-D model, which include both inputs and measured values upon convergence, is observed to be within acceptable ranges for general human population (Asmar et al. 2001; Phan et al. 2017). Furthermore, extreme cases within physiologically acceptable range for each parameter, as shown in minimum and maximum values, have also been generated and recorded in the database. These cases allow for the neural networks, upon which the objective of present work is primarily based on, to be trained on a wide variety of physiologically possible cases for achieving higher accuracies.

**Table 4:** Baseline characteristics (average, 95% CI in brackets) of virtual patients (with AAA) used in the virtual database

	Small AAA [$n=1958$] ($\hbox {Females}=915$)	Medium AAA [$n=1164$] ($\hbox {Females}=455$)	Large AAA [$n=1400$] ($\hbox {Females}=436$)
Age (years)	67.29 [66.70–67.87]	74.40 [73.81–74.98]	77.80 [77.47–78.13]
Weight (kg)	95.47 [94.74–96.19]	97.25 [96.3–98.21]	87.71 [87.13–88.29]
Height (cm)	174.18 [173.49–174.87]	176.11 [175.20–177.02]	177.95 [177.20–178.70]
Ejection fraction (%)	61.85 [61.50–62.21]	62.45 [62.00–62.89]	60.44 [60.22–60.66]
Cardiac output (L/min)	4.72 [4.67–4.77]	4.68 [4.62–4.74]	6.77 [6.70–6.83]
Pulse pressure (mmHg)	42.04 [41.45–42.63]	42.73 [42.02–43.44]	70.53 [69.76–71.29]
Pulse wave velocity (m/s)	8.97 [8.92–9.02]	9.45 [9.39–9.50]	10.54 [10.51–10.57]
AAA diameter (cm)	3.97 [3.96–3.98]	4.98 [4.96—4.99]	5.97 [5.95–5.98]
AAA length (cm)	5.31 [5.27–5.35]	5.79 [5.74–5.83]	7.75 [7.71–7.80]

Table 4 shows the average values (with 95% confidence interval) of key parameters in AAA virtual patients. The maximum and minimum input values for the AAA patients are similar to that of non-AAA patients (see Table 3). Values in Tables 3 and 4 are chosen for close physiological representation of general human population. However, since several approximations and assumptions are made in the 1-D model, a lot of features may be lost with respect to real human patient data. Thus, any system trained on these databases must be bolstered with real human data, using transfer learning or other methods before being tested in clinical settings.

### Inverse analysis using neural networks

As mentioned previously, inverse analysis is performed to predict blood pressure waveforms at various locations in the arterial network using virtual measurement at peripheral arteries. The inverse analysis model developed using LSTM-based neural network, during training, calculated blood pressure waveforms at arteries located upstream to the points of input measurement, such as aortic root and abdominal aorta, with an acceptable level of accuracy. The results from this model are analysed primarily for two aspects. Firstly, its capacity to predict important features of the waveform such as dicrotic notch in aortic root. Secondly, the accuracy with which the blood pressure values at each time step is calculated. In order to explain different aspects of this analysis, results from one healthy virtual subject (from the virtual patient database) are described in this section.

**Fig. 11:**
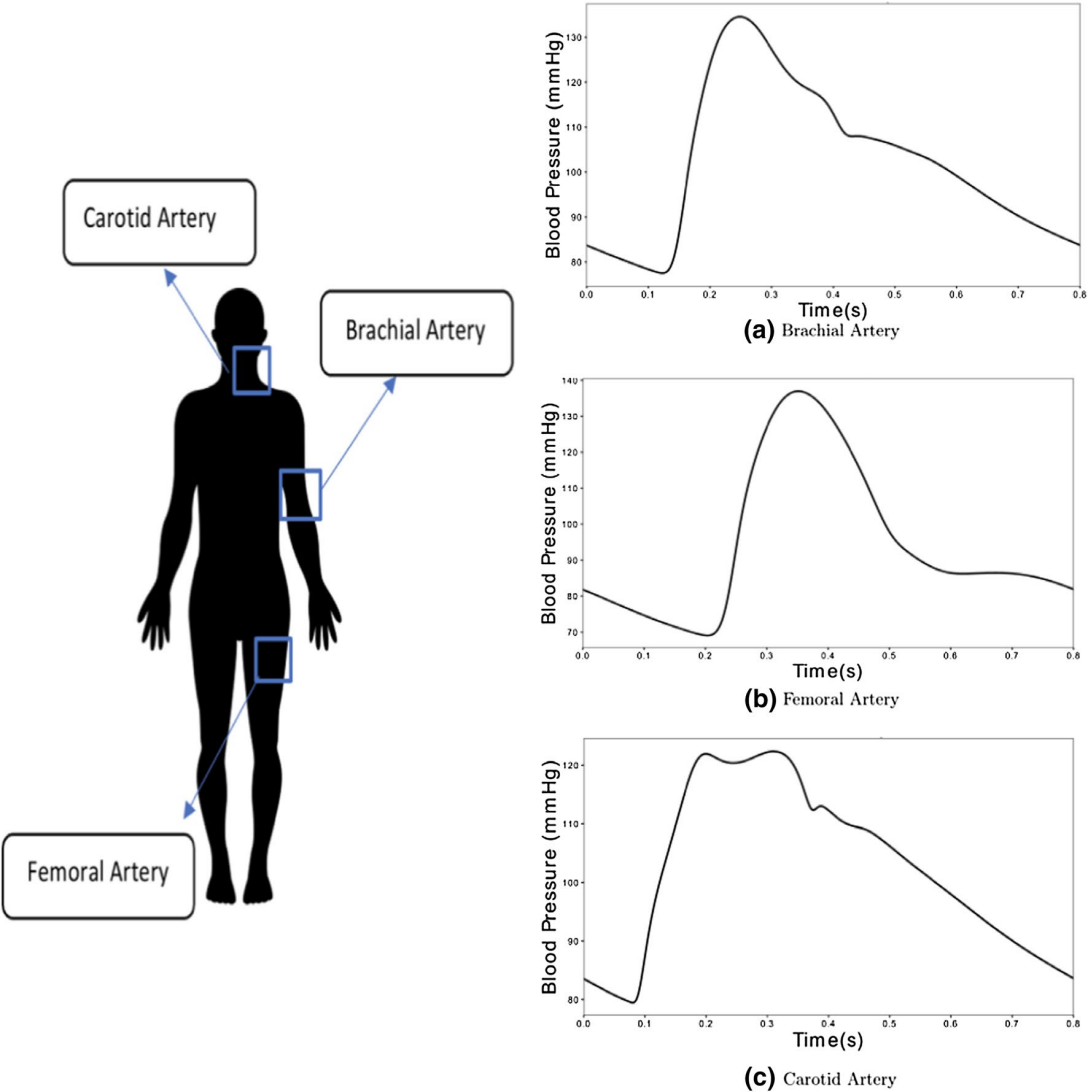
Input blood pressure waveforms measured at three different locations

The input blood pressure waveforms are measured in carotid, femoral and brachial arteries as shown in Fig. 11. These waveforms are obtained from the haemodynamic model of a virtual subject. The measurements are taken from nodes located midway along the length of the arteries.

**Fig. 12:**
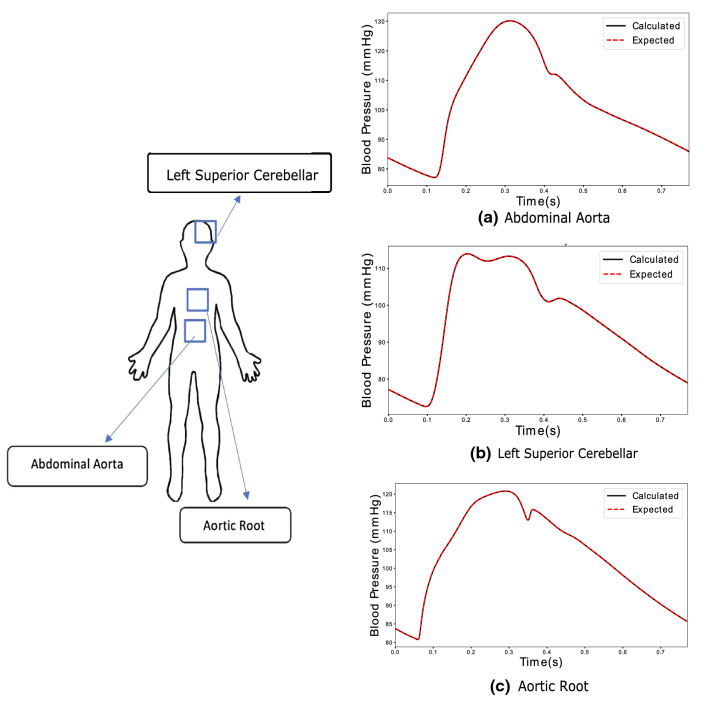
Outputs calculated for inputs in Fig. 11 by RNN in healthy subjects at different locations

**Fig. 13:**
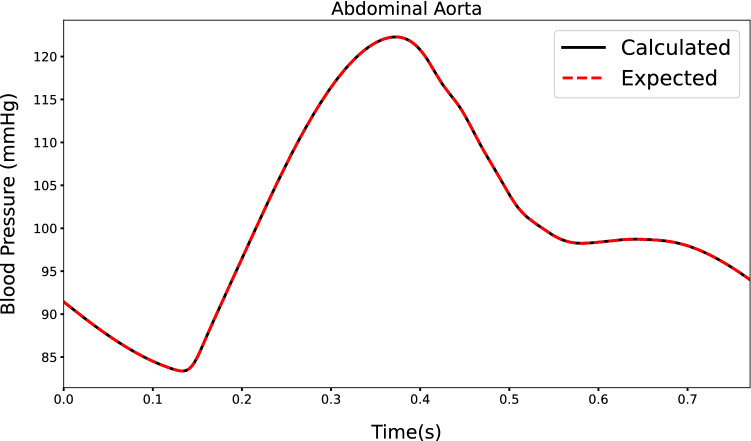
Output calculated at the distal end of abdominal aorta by LSTM-based neural network for inverse analysis in a human patient for large AAA condition

Figure 12 shows the waveforms at abdominal aorta, left cerebral artery and aortic root. Both calculated (solid line) and expected (dotted line) are shown. The dicrotic notch (occurring at approximately 0.36 s), peak pressure (occurring at approximately 0.25 s), minimum pressure (at the start of the cardiac cycle) and shape of waveform for different arteries are calculated. As seen, both calculated and expected pressure waveforms agree excellently apart from minor differences observed in the aortic root. This accuracy extends also to the case of a virtual patient suffering from AAA (see Fig. 13). The only significant deviation found in these output waveforms, which is also observed in other virtual patients, occurs slightly before the dicrotic notch.

When observed on the entire virtual patient database, proposed procedure performs with promising results. Blood pressure waveforms in arteries upstream to the input vessels (carotid, brachial and femoral) such as aortic root, abdominal aorta and iliac arteries (see Fig. 12) are calculated with an acceptable lowest accuracy of 98.81%, amongst the arteries. As mentioned in 2.3.1, the data is split into 80% for training data set and 20% testing data set. The lowest accuracy observed amongst the waveforms predicted for testing data set, which is not used during training, is 94.16%. An acceptable accuracy is assumed when averaged error between expected and calculated pressure waveforms over a cardiac cycle is less than 0.5 mmHg.

### Application of inverse analysis to the classification of aortic abdominal aneurysm (AAA)

In this section, convolutional neural network (CNN) is used to classify AAA severity based on diameter into categories [Healthy (< 3cm), small AAA (3–4.4 cm), medium AAA (4.5–5.4 cm), large AAA (> 5.5 cm)], with an accuracy of 97.79%, when observed over the entire database. On testing data set, data not used for training, network is observed to perform with an accuracy of 90.58%.

**Fig. 14:**
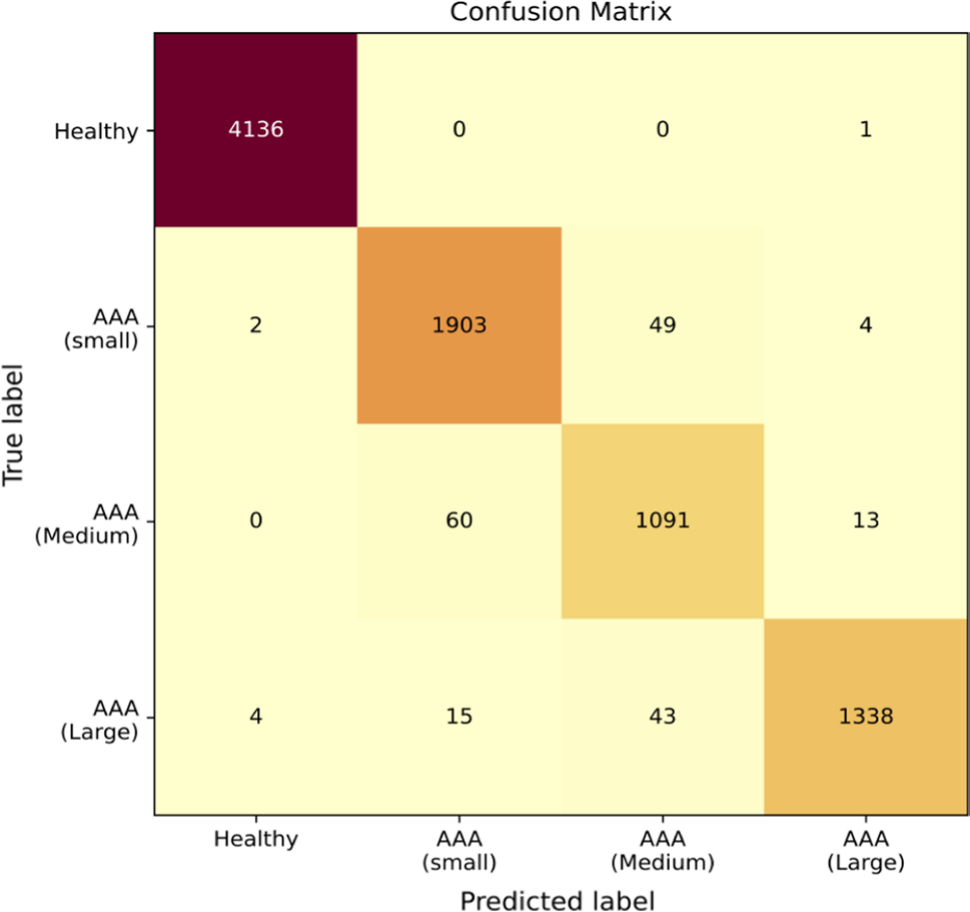
Confusion matrix to understand the performance of the model for virtual patients in the entire database

Characteristics such as relatively higher pressure in femoral artery compared to brachial artery in healthy patients, a contrast to AAA cases, could be one of the important features picked up by the CNN using pressure waveform calculated at the distal end of abdominal aorta. The CNN, using convolutional filters in cascaded nonlinear domains, captures various features of this waveform to detect AAA and classify its severity. Figure 14, a confusion matrix, displays the performance of the classifier with respect to multi-classification (Pedregosa et al. 2011).

In the confusion matrix (Fig. 14), it can be observed that true positives ( 4332 patients) and true negatives (4136 patients) make up the highest number of outputs calculated, making it a potential system with high reliability.

Furthermore, when only detection of AAA and not its severity to be considered, close to ideal performance of 99.91% accuracy can be observed. It must be noted that in Fig. 14, all miss-classified cases below their actual severity (lower diagonal) are treated as false negatives and the ones above (upper diagonal) as false positives. However, when only detection of AAA is considered, only the cases miss-classified as healthy are considered as false negatives. With very low number of false negatives, proposed method may be seen as a reliable method when used as a detector of aneurysms.

## Conclusions

The proposed approach of inverse analysis makes development of an active digital twin, capable of continuously monitoring, and preventing medical conditions from developing or further aggravating feasible. This approach for biomedical applications with the help of non-invasive or minimally invasive measuring tools has the potential to reduce dependencies on sophisticated and invasive diagnostic tools.

In the case of a cardiovascular system, this approach is potentially implementable in clinical environments and aids in monitoring critical vessels and cardiovascular parameters. The method presented in this work can perform inverse analysis with high accuracy. It detected problems such as AAA with an accuracy as high as 99.91% and classified its severity with acceptable accuracy of 97.79%. With these results obtained in this work, it can be concluded that this approach may be used to monitor parameters with on-par accuracy to that of conventional diagnostic tools if the real system behaves according to the physics employed in the present work.

However, two key issues still need to be resolved before deployment in clinical environments. Firstly, since only one cardiovascular condition is modelled, the possibility of multiple other conditions or diseases generating the same output waveforms still exist. This is a common problem faced in inverse analysis, where multiple solutions for a given problem are possible. This work proposes the idea of using deep learning technique for inverse analysis in biomedical applications. However, a solution of using probability distribution for identifying the cause, when multiple clinical conditions give rise to the same output, needs to be developed to avoid false diagnosis.

Secondly, since the deep learning model is trained on a virtual patient database, possibility of decrease in accuracy of the system is possible when exposed to clinical environments. To avoid this, the system must be additionally trained using transfer learning on real human patient data.
